# Reliability and Lifetime Assessment of Glider Wing’s Composite Spar through Accelerated Fatigue Life Testing

**DOI:** 10.3390/ma13102310

**Published:** 2020-05-17

**Authors:** Sebastian Marian Zaharia, Mihai Alin Pop, Răzvan Udroiu

**Affiliations:** 1 Manufacturing Engineering Department, Transilvania University of Brasov, Eroilor 29 Str, 500036 Brasov, Romania; udroiu.r@unitbv.ro; 2 Materials Science Department, Transilvania University of Brasov, Eroilor 29 Str, 500036 Brasov, Romania; mihai.pop@unitbv.ro

**Keywords:** composite spar, accelerated fatigue life testing, reliability, lifetime, glider

## Abstract

The evaluation of the reliability and the lifetime of aerospace components has become an important segment of the design stage. The aeronautical components are subjected to complex, rigorous tests and have a long test life. The main goal in the field of aviation is to have components with high reliability and quality and to meet the mandatory requirements and regulations. The spars are stiffening components positioned along the wing and which take up most of the load and are tested for fatigue over a long period of time. The spar which was analysed in this study has a sandwich structure with GFRP (glass fiber reinforced plastic) skin and foam core. In this paper, the performances in the static and dynamic conditions of the GFRP-foam sandwich structures cut out of the composite spar of a glider were analysed. Additionally, using accelerated techniques based on the three-point fatigue bending test, the main reliability indicators of the GFRP-foam sandwich structures were determined. Using the statistical processing of the experimental data and the Inverse Power Law–Weibull acceleration model, the mean number of cycles to failure, in normal testing conditions of the GFRP-foam specimens was determined, with a value of 102,814. Using the accelerated testing techniques of the GFRP-foam sandwich structures an important decrease of the test time (8.43 times) was obtained.

## 1. Introduction

Traditionally, the analysis of the behaviour while operating aerospace components is made based on their lifetime, which is obtained by following up the functioning of the components under normal operating conditions. In many situations, however, deducting the lifetime of aerospace systems, within a reasonable amount of time, is difficult or even impossible, to achieve for various reasons, such as [1] the very long lifetime in the case of highly reliable systems, which, in some situations, can be of the order of years; the very short time between the design and the manufacturing stages; and the continuous change of the test conditions in which normal operating regimes are used.

Due to these difficulties, the engineers in the field of experimental reliability have generated a series of methods to force the occurrence of the failure mechanisms of the aerospace systems, in order to produce the failures having as a consequence the reduction of the testing time. Over the years, these practices have materialized through well-defined tests, called accelerated life testing. There are a variety of methods that align under the name of accelerated life testing, all with the ultimate goal of deducing the operating characteristics and reliability indicators of the aerospace systems under normal operating conditions. Accelerated or forced tests involve stressing the aerospace systems tested at higher stress regimes than those of normal operation [2].

Studies on accelerated life testing can be divided into several main directions: developing hypotheses and statistical models necessary for estimating reliability indicators and confidence intervals [3]; optimization of models and accelerated test plans [4,5,6]; accelerated fatigue tests used on different industrial materials and products [7,8,9,10,11,12]; and the extension of the scope of application of accelerated testing techniques on various industrial products [13,14,15,16,17]. The extremely varied and complex stresses applied to the aerospace components have led to experimental studies, in order to ensure the proper functioning and the required reliability under maximum stress regime, at a minimum material and labour use. The methods of estimating the reliability of the aerospace components [18,19] take, also, into account, on the one hand, the actual structural properties of the materials from which they are manufactured, and on the other, it is necessary to carry out tests in a spectrum of stresses as similar to the actual operating conditions.

The use of accelerated life testing in the aerospace field was imperative because most components, such as bearings [20], landing gear [21], pitch-change-link [22], helicopter blade [23], and helicopter airframe [24], present a long lifetime. Thus, if an aerospace component requires, for example, 10^6^–10^7^ cycles to produce a fatigue failure in normal testing conditions, by using accelerated life testing the same result can be obtained after 10^4^–10^5^ cycles. Therefore, more in-depth studies on fatigue behaviour have been carried out using accelerated life testing in the aerospace field on various components, such as the helicopter’s mission system sensor-cowling assembly [25], pitch links [26], helicopter blade [27], and supple platinum [28]. In these studies, based on aerospace components, the theory was validated that accelerated techniques result in a significant reduction in testing time and material costs, determining in the shortest possible time the lifetime and the main reliability indicators. Intense research, on fatigue behaviour [29,30,31] and simulation with the finite element method [32,33,34] have been carried out, in recent years, for the spar of the wings.

The purpose of this paper is to study the behaviour and the performances in static and dynamic regime of sandwich samples cut from the main spar web from a glider made of composite materials. The GFRP (glass fibre reinforced plastic)-foam sandwich structures were static three-point bending tested and the main mechanical properties (bending strength and bending modulus) were determined. For the dynamic regime, accelerated reliability techniques were introduced, where the testing regime of sandwich structures is accelerated, by increasing the loading frequency. Using this acceleration methodology, the mean lifetime and reliability indicators for GFRP-foam sandwich specimens can be determined in a short time.

## 2. Materials and Methods 

**Figure 1:**
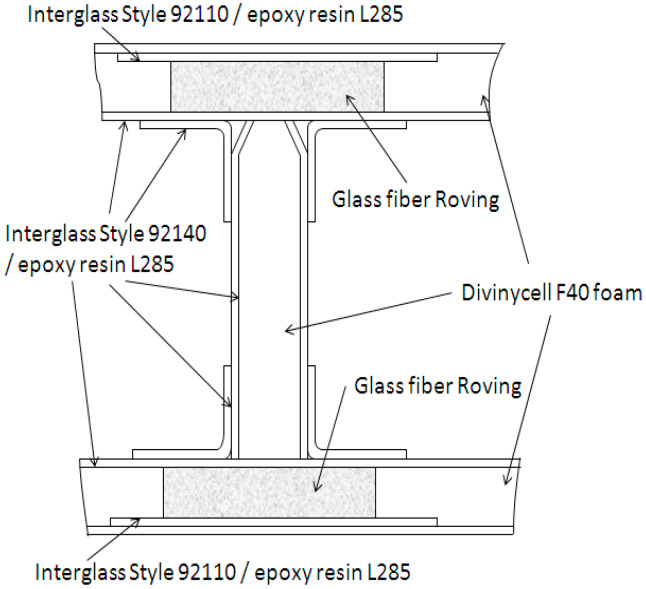
Presentation of the materials from which the composite spar is manufactured [35].

The structure of an experimental glider built in Brasov, Romania was entirely made from composite materials. The samples which were tested in this paper were extracted from the main spar web of the glider wing. The wing is a composite structure mainly made of glass fibre and thermosetting resin (Figure 1).

The main spar web presents a sandwich structure with a foamed plastic core covered by two face sheets bonded on it. The spar web core is made of Divinycell F40 material from DIAB Group (Helsingborg, Sweden). The upper and lower spar cap is built from glass fiber roving. Divinycell F foam is a recyclable, sandwich core, offering excellent fire, smoke and toxicity properties, and good mechanical and processing characteristics, such as exceptional fatigue life and good chemical resistance [36]. The face sheets of the spar web are made of one layer of Interglas Style 92140, Twill 2/2 weave pattern from Interglass Textil Gmbh [37] and mixed epoxy resin/hardener (L285/H286). The wing components are fabricated by wet layup in a mold and then vacuum bag cured.

For static and fatigue three-point bending tests of the GFRP-foam sandwich specimens, 30 specimens were cut from the structure of the composite spar. Thus, 10 specimens were static three-point bending tested in accordance with ASTM C393 standard. The universal WDW-150S machine (Jinan Testing Equipment IE Corporation, Jinan, China) was used for static testing of GFRP-foam specimens and the crosshead speed was 2 mm/min. The GFRP-foam sandwich structures were positioned on the supporting pins and the span length was 110 mm. 

The main scope of the three-point bending tests in static regime was to determine the mechanical performance (the bending strength the elastic modulus, the core shear ultimate strength, the core shear yield stress and facing stresses) of the GFRP-foam sandwich structures. 

The dimensions of the specimens, static and fatigue three-point bending tested are presented in Table 1.

**Figure 2:**
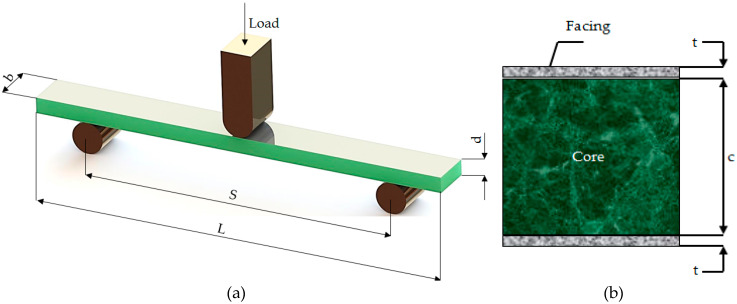
Principle of the three-point bending test of the GFRP-foam specimens (**a**). Cross section of the GFRP-foam specimens (**b**).

The physical model used in the three-point bending test of GFRP-foam sandwich specimens is described in Figure 2a. Figure 2b shows a cross-sectional view of the tested sandwich structure.

Equations (1) and (2) were used in order to determine the values for bending strength (σ_b_) and bending modulus (E_b_), specific to sandwich structures subjected to three-point bending testing:

(1) $$\sigma_{b}=\frac{3\mathrm{PS}}{{2\mathrm{bd}}^{2}}$$

(2) $$E_{b}=\frac{S^{3}m}{{4\mathrm{bd}}^{3}}$$

where P is the load (force) at a given point on the load deflection curve (N), S is the length of support span (mm), b is the sandwich specimen width (mm), d is the sandwich specimen thickness (mm) and m is the slope of the tangent to the initial straight line portion of the load-deflection curve.

The ASTM C393 standard provides the equations for the calculation of the following mechanical characteristics, specific to three-point bending tested sandwich structures: the core shear ultimate strength, τ_csu_ (Equation (3)), the core shear yield stress, τ_cy_ (Equation (4)) and facing, σ_f_ (Equation (5)):

(3) $$\tau_{\mathrm{csu}}=\frac{P}{(d+c)b}$$

(4) $$\tau_{\mathrm{cy}}=\frac{P_{y}}{(d+c)b}$$

(5) $$\sigma_{f}=\frac{\mathrm{PS}}{2t(d+c)b}$$

where c is the core foam thickness (mm), P_y_ is the core foam shear yield stress (MPa) and t is the skin thickness (mm).

For fatigue three-point bending tests, 20 specimens were cut from the sandwich GFRP-foam structure of the spar. The test methodology for these specimens was in accordance with MIL-STD-401B Sec. 5.3. Accelerated fatigue tests of the samples were conducted at four frequency loading levels: 2 Hz, 3 Hz, 4 Hz and 5 Hz, using a WDW-150S universal testing machine. The accelerated fatigue tests were conducted at room temperature in load control, at 2 Hz, 3 Hz, 4 Hz and 5 Hz under sinusoidal cyclic loading and with a minimum-to-maximum stress ratio of R = 0.1. Accelerated fatigue data of the GFRP-foam sandwich specimens were generated at a load level of 50% ultimate static load.

## 3. Results and Discussion

### 3.1. Static Mechanical Proprieties

**Figure 3:**
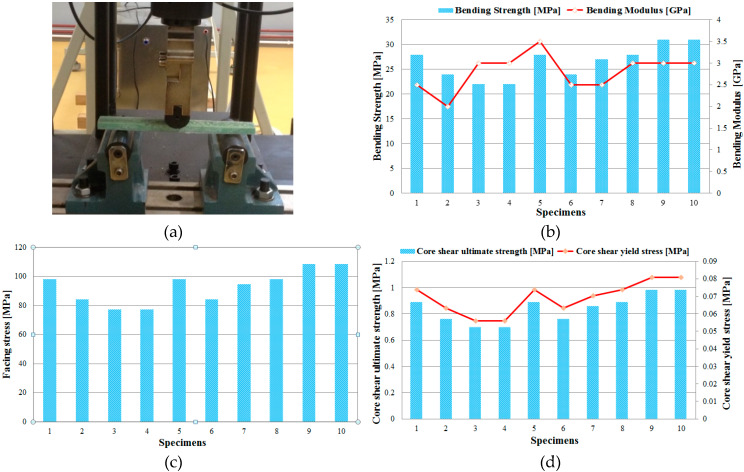
Three-point bending tests of the sandwich GFRP-foam specimens: (**a**) Test setup for three-point bending; (**b**) bending strengths and modulus of the GFRP-foam sandwich specimens; (**c**) facing stresses of the GFRP-foam sandwich specimens; (**d**) core shear ultimate strength and core shear yield stress of the GFRP-foam sandwich specimens.

The static three-point bending tests were performed on ten GFRP-foam samples, cut from the structure of the spar of the glider made from composite materials, until their breakage occurred. Using a WDW-150S test machine (Figure 3a) program, the important mechanical properties (bending strength, bending modulus, core shear ultimate strength; core shear yield stress and facing stress) of the analysed sandwich structures were determined (Figure 3b). Using Equations (1) and (2) and the dimensions of the sandwich specimens, the test machine software automatically determines the main three-point bending characteristics (the bending strength and the bending modulus). The three main characteristics (the core shear ultimate strength, τ_csu_, the core shear yield stress, τ_cy_ and facing stress, σ_f_) described in the ASTM C393 standard were calculated and plotted in Figure 3c,d.

For each data series (bending strength, bending modulus, core shear ultimate strength; core shear yield stress and facing stress) obtained from the three-point bending tests, the statistical indicators (mean, standard deviation, coefficient of variation) were determined for the GFRP-foam sandwich specimens, according to the statistical relationships provided in the ASTM C393 standard. For the above data series, the coefficient of variation was calculated so as to have a clear picture of the homogeneity of the experimental data. From the results described in Table 2 it can be seen that the coefficient of variation has values between 12.452% and 14.285% and it can be estimated that the mean is representative for the five sets of experimental data.

**Figure 4:**
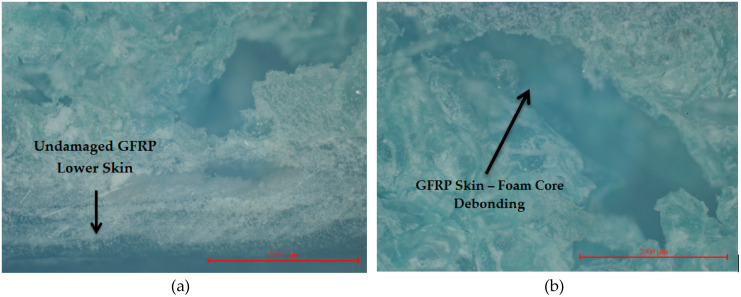

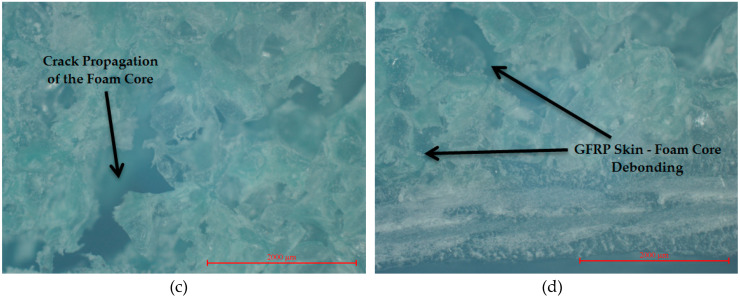
Macroscopic views (magnification 25×) of the crack propagation during the three-point bending tests of the (**a**) GFRP-foam specimen; (**b**) Upper GFRP skin-foam core debonding; (**c**) crack propagation of the foam core; (**d**) lower skin-foam core debonding.

Optical micrographs were performed using a Nikon Eclipse MA100 (Nikon, Tokyo, Japan) inverted light microscope. The three-point bending tests of the GFRP-foam specimens showed that the skins did not suffer major damage, remaining intact, maintaining their original characteristics (Figure 4a). The failure mode of the entire sandwich structure was mainly determined by the phenomenon of rapid shearing of the foam core, which was followed by crushing and creating holes in the foam core. Due to the stress concentration of the foam core, crack propagation occurs rapidly with the increase of the three-point bending load and finally, it causes structural damage of the whole core. In most bending tested structures, the debonding of GFRP skins from the foam core was observed. Regarding the way the debonding occurs, for the tested specimens it was observed that the foam adhered to the GFRP skin, which indicates that the bonding between the core and the skins of the sandwich structure is very effective. The first phase (Figure 4b) consists of the debonding of the GFRP skin in the middle area of the sandwich structure, where the force was applied, on the upper skin that is subjected to compression. A second phase consists of the onset of the crack of the foam core (Figure 4c) and the subsequent propagation of the crack to the lower skin (Figure 4d). Due to the same raw materials and the same manufacturing technology were used in the manufacture of the sandwich structures, it can be stated that the tested specimens showed a similar failure behaviour as a result of the three-point bending tests. From the main failure mode (core shear) of the GFRP-foam sandwich structures it can be deduced that the skins are well sized, thick enough and strong and they remain unaffected after the bending tests are carried out.

### 3.2. Accelerated Fatigue Life Testing of the GFRP-Foam Specimens

Under the current conditions of aerospace safety, static tests of composite sandwich structures must be duplicated by dynamic fatigue tests. Since composite sandwich structures have a long lifetime accelerated fatigue life testing was used in this study. Thus, in this paper, accelerated fatigue life tests on GFRP-foam sandwich specimens were proposed, which consisted in increasing the loading frequency (2 Hz, 3 Hz, 4 Hz and 5 Hz) compared to normal testing conditions (1.5 Hz), in order to intensify the degradation processes. A normal testing condition for composite materials and composite sandwich structures is performed at a loading frequency of 1.5 Hz, according to previous studies [7,38,39,40]. In the accelerated fatigue life tests, 20 samples were tested at four levels of accelerated testing, at the following loading frequencies: 2 Hz, 3 Hz, 4 Hz and 5 Hz. Using the acceleration methodology, the number of cycles to failure for each sandwich specimen was obtained using the accelerated fatigue life tests (Table 3).

### 3.3. Reliability Analysis of Accelerated Life-Test Data

**Figure 5:**
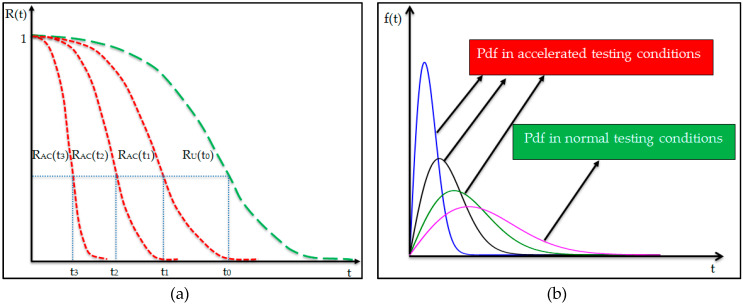
Equivalence of accelerated tests: (**a**) Variation of reliability under accelerated test conditions and in normal testing conditions; (**b**) variation of probability density in accelerated testing conditions and in normal testing conditions.

The main drawback of the reliability tests carried out in normal test level is represented by the long duration of these tests. The solution to reducing the testing time is to accelerate the applied stresses. An important aspect in carrying-out the accelerated tests is the equivalence between accelerated tests and normal tests. The equivalence relation is obtained on the basis of the postulate of equal reliability which constitutes the theoretical basis of these types of tests. Figure 5a shows three stress levels under accelerated condition which correspond to the three reliability functions (R_AC_ (t_3_), R_AC_ (t_2_) and R_AC_ (t_1_)) and the stress level in normal test condition with the reliability function (R_U_(t_0_)). The postulate of equal reliability is expressed by Equation (6).

(6) $$R_{\mathrm{AC}}\left(t3\right)=R_{\mathrm{AC}}\left(t2\right)=R_{\mathrm{AC}}\left(t1\right)=R_{U}\left(t0\right)$$

**Figure 6:**
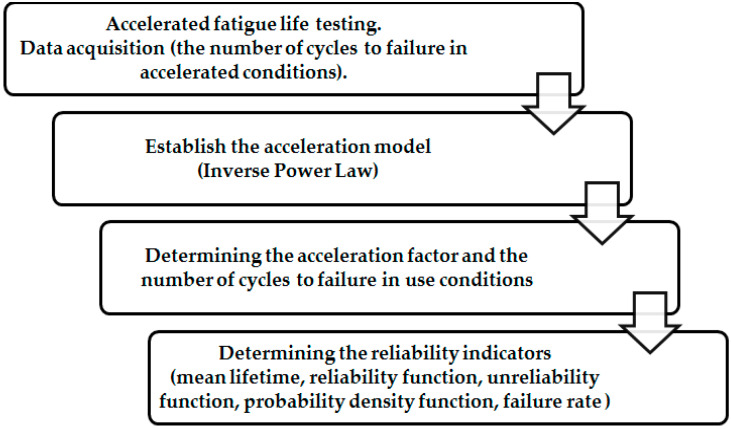
The statistical processing algorithm of the GFRP-foam specimens.

Also, an equivalence relation can be established for the Weibull statistical distribution in terms of probability density function (pdf). Figure 5b shows four forms of the probability density of the Weibull distribution, where the shape parameter has the same value (β = 2), for all distributions, and the scale parameter η will have different values: for accelerated test conditions (η_3_ = 100, η_2_ = 200 and η_1_ = 300), and for normal testing conditions (η_0_ = 400). Therefore, for the validation of accelerated tests, the probability density function under accelerated test conditions must be in the same shape as that found in normal testing conditions. In other words, for the equivalence of accelerated tests, the shape parameter must have the same value, both under accelerated and normal testing conditions. As for the scale parameter, it has different values under accelerated testing conditions as well as in normal testing conditions. Figure 6 describes the methodology of statistical processing of GFRP-foam sandwich specimens subjected to accelerated life tests.

Accelerated life test involves measuring the performance of aerospace components under accelerated stress conditions, than in normal operation, at different acceleration levels. This technique determines the reduction of the lifetime of the aerospace components, by intensifying their failure mechanisms. After collecting the data from the accelerated regime, corresponding to the different levels of acceleration, using specific mathematical models (acceleration models), the lifetime of the aerospace component under normal testing conditions can be determined, materialized by an appropriate distribution.

The extrapolation technique was used in order to make the connection between the accelerated test data and the normal test data. Thus, the accelerated test results were extrapolated to the normal test level of 1.5 Hz. Accelerated tests showed four levels of acceleration (2 Hz, 3 Hz, 4 Hz and 5 Hz), and for each level five GFRP-foam sandwich specimens were tested. Using accelerated techniques, the number of cycles to failure, for each tested GFRP-foam sandwich sample was obtained, using the fatigue three-point bending testing. Data from the accelerated tests (number of cycles to failure) were entered into the ALTA 7, Accelerated Life Testing Analysis software [41], and they were extrapolated, using the inverse power law model and the Weibull distribution, to the normal test level of 1.5 Hz.

For statistical processing, the inverse power law (IPL)-Weibull model was used. This model is best applicable for the techniques of accelerating the aerospace structures where the failure mode is represented by the fatigue phenomenon. The three parameters characteristic to IPL-Weibull acceleration model are determined by the maximum likelihood estimation method for the accelerated data information, obtaining the following values: β = 2.496; k = 2.366 × 10^−6^; n = 3.191. The number of cycles to failure in normal testing conditions of GFRP-foam sandwich specimens (Table 4) is obtained as the product of the number of cycles to failure under accelerated test conditions and the acceleration factor.

The study of the reliability of an aerospace component consists in the analysis of the elapsed lifetime since the component is brought into operation until its failure. This lifetime is of random length and its numerical characteristics are called reliability indicators. The reliability indicators characterize quantitatively the reliability of the aerospace components.

In this paper, a sample of 20 GFRP-foam sandwich specimens was analysed, which was subjected to a single stress (three-point bending) at a constant level of acceleration, for which a series of operating times data was collected (the number of cycles to failure in normal testing conditions). Based on the number of cycles to failure in normal testing conditions, by the specific statistical processing, the probability density distribution can be deduced and, consequently, a series of reliability indicators. Frequently, the mean time to failure, MTTF, is determined. The objective of the accelerated test is not only to obtain predictions regarding the behaviour of the aerospace component in accelerated conditions, but also to other conditions, empirically, in the normal testing conditions.

Table 5 presents the dependence between the number of cycles to failure in normal testing conditions and the main important reliability indicators (reliability function, unreliability function, probability density function—pdf and the failure rate) of the GFRP-foam sandwich structures. The reliability indicators in normal testing conditions (normal testing level of the frequency—1.5 Hz) of the GFRP-foam specimens are determined using equations specific to the IPL-Weibull model according to the number of cycles until failure.

For the graphical representation of the reliability indicators, the three-dimensional model was chosen, for which a surface that presents the dependency between the reliability indicators (reliability function, unreliability function, probability density function and failure rate) time-stress level was chosen.

**Figure 7:**
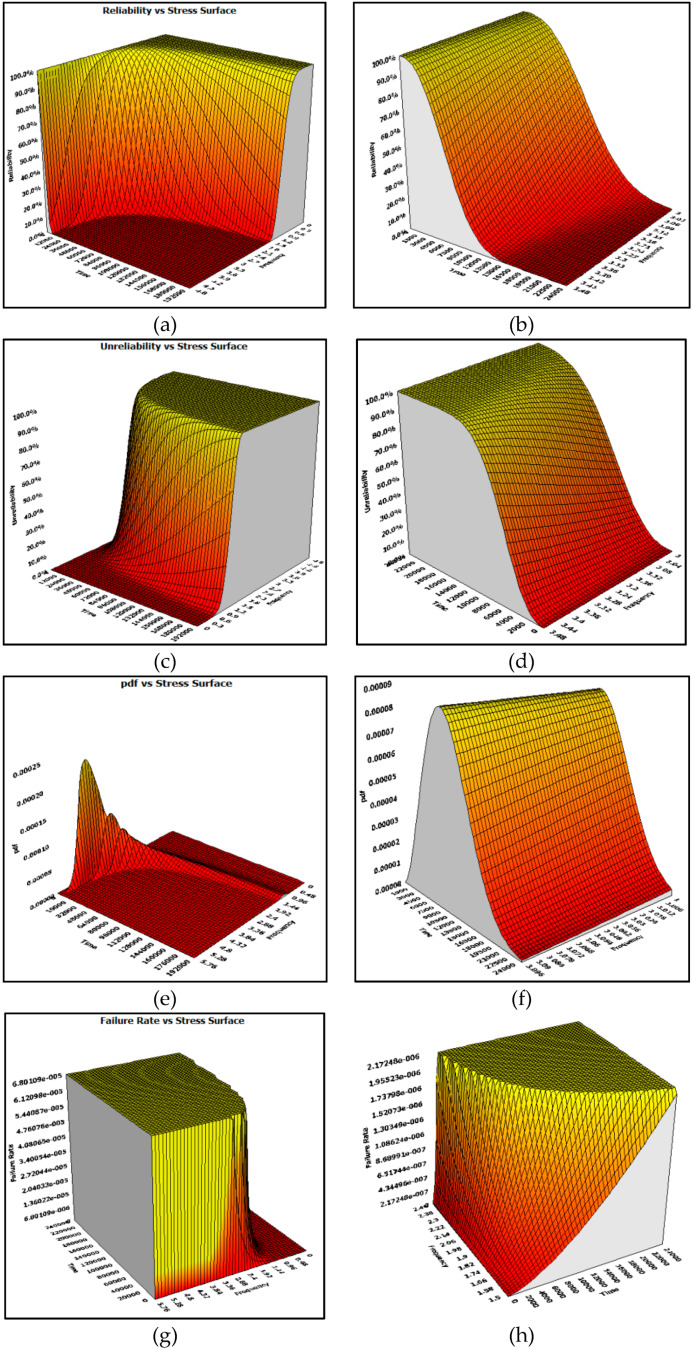
The reliability indicators of the GFRP-foam sandwich specimens: (**a**) Reliability function; (**b**) local amplification of the reliability function; (**c**) unreliability function; (**d**) local amplification of the unreliability function; (**e**) probability density function; (**f**) local amplification of the probability density function; (**g**) failure rate; (**h**) local amplification of the failure rate.

In the Figure 7a there is a 3D representation of the reliability–time-frequency. The reliability function is a quantitative reliability indicator and presents an important practical utility in the study of the accelerated life tests. Through this function the lifetime of an aerospace component for a certain value of reliability and stress (frequency, in this case study) can be determined. Figure 7b shows the three-dimensional reliability function with a local amplification in the following range: frequency between 3 and 3.5 Hz and time between 0 and 25,000 cycles to failure. The unreliability function represents the probability that the GFRP-foam sandwich structures will fail before a pre-set time. Additionally, from the 3D graph of the unreliability function (Figure 7c), useful information regarding the number of cycles to failure in normal testing conditions of use of the GFRP-foam sandwich structures can be obtained, depending on the unreliability and stress. A local amplification of the 3D graphic representation of the unreliability function (Figure 7d) was crated as follows: frequency between 3 and 3.5 Hz and time between 0 and 25,000 cycles to failure. The behaviour of the aerospace component around a given moment is described by the given probability density function. The 3D graphical representation of the probability density function of the GFRP-foam sandwich structures (Figure 7e) is made based on the data regarding the moments of occurrence of the failures according to the distribution law governing the given process. The probability density graph (Figure 5f) was detailed using a local amplification in the range: frequency between 3 and 3.5 Hz and time between 0 and 25,000 cycles to failure. The failure rate of aerospace components is a feature used to estimate the reliability of aerospace systems. From a statistical point of view, the failure rate is equal to the ratio between the number of failures that occur in a unit of time and the number of components that are still functioning until that moment. Figure 5g describes the behaviour of the failure rate of the GFRP-foam sandwich structures according to the number of cycles to failure in normal testing conditions and the stress level. For a more explicit graphical representation of the three-dimensional failure rate, a local amplification (Figure 7h) was performed in the frequency range between 1.5 and 2.5 Hz and time between 0 and 25,000 cycles to failure.

It is useful to know the fatigue behaviour of the aerospace components and, therefore, their lifetime because the most suitable times for replacing them can be chosen in order to obtain the lowest maintenance cost and to have maximum operating safety.

**Figure 8:**
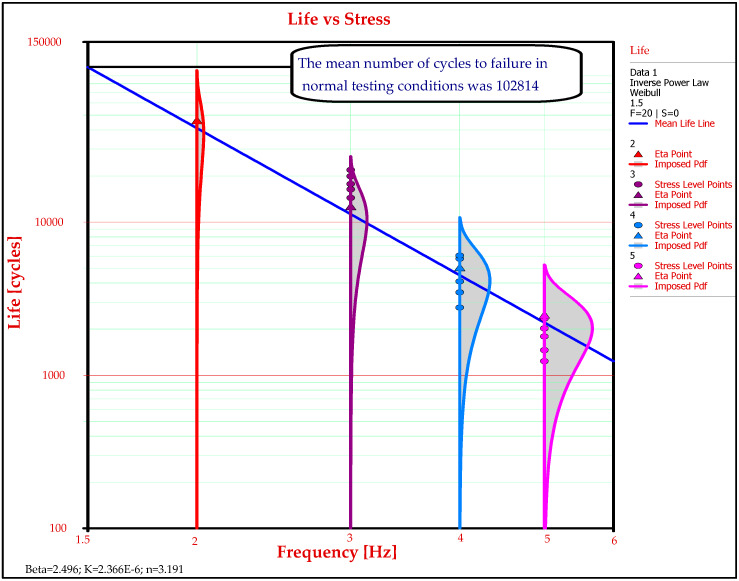
Determining the mean number of cycles to failure of the GFRP-foam sandwich specimens in normal testing conditions.

The mean life assessment for the GFRP-foam sandwich specimens represents one of the main objectives of this paper. In order to determine the life characteristic specific to the Weibull distribution, the graphical method is used. The mean number of cycles in normal testing conditions estimate the time at which 63.2% of the tested GFRP-foam sandwich specimens are expected to fail for the four accelerated levels (2, 3, 4 and 5 Hz). At the intersection of the Eta curve, which estimates the mean life (63.2%), with the axis from the normal stress level (frequency) of 1.5 Hz, finds itself the mean number of cycles to failure in normal testing conditions of the GFRP-foam sandwich specimens, which is 102,814 (Figure 8).

## 4. Conclusions

The use of the reliability concept in the field of the mechanics of composite materials and the behaviour of aerospace structures is a problem of special complexity and importance, which is given permanent attention. The application of the concept of reliability in engineering in general, and in aircraft construction, in particular, is an increasingly obvious necessity, aiming from both technical and economic considerations the transformation of the notion of reliability into one of the main characteristics of an aerospace product.

The sandwich structures investigated in this paper were cut from a composite web spar of a glider. Furthermore, the mechanical properties of GFRP-foam sandwich specimens subjected to static and dynamic three-point bending tests were determined. From the analysis of statistical data from static compression tests of GFRP-foam specimens it can be seen that the limit threshold (δ% > 30%) from the state of homogeneity to the state of heterogeneity is not exceeded, so the data are representative for the studied sample. The compressive strength of GFRP-foam specimens is between 22 and 31 MPa, and the modulus of elasticity is between 2 and 3.5 GPa. The microscopic analysis of the failure mode of GFRP-foam specimens showed a debonding of the foam from the upper skin followed by a crack propagated throughout the core, and then a debonding from the lower skin. Since the test duration of the aerospace components is very long, the use of accelerated techniques is chosen. The methodology of accelerated fatigue testing, at four levels (frequencies) of accelerated stress using three-point bending tests of sandwich specimens, was used in this paper in order to determine their failure mode.

By accelerating the loading frequency level, the main reliability indicators of GFRP-foam sandwich specimens, under normal testing conditions were determined and represented graphically. The information obtained from the accelerated three-point bending tests signalled an important aspect, namely, by increasing the loading frequency, the performance on breaking strength and lifetime of GFRP-foam sandwich specimens is reduced. This paper presents a reliability and lifetime assessment of aerospace component (composite wing spar) using the accelerated test approach. Using the accelerated tests, the mean fatigue lifetime (102,814 cycles) was calculated, in normal testing conditions of GFRP-foam specimens using statistical processing and calculation relationships specific to the inverse power law-Weibull model. The acceleration techniques of the experiments are used to determine reliability indicators as quickly as possible and to predict the fatigue lifetime of the aerospace products.

## Figures and Tables

**Table 1:** Dimensions of sandwich specimens tested in three-point static and fatigue bending.

Material	Length L (mm)	Thickness d (mm)	Width b (mm)	Span Length S (mm)	Core Thickness c (mm)	Facing Thickness t (mm)
GFRP-Foam	150	10	15	110	9	0.5

**Table 2:** Statistical indicators determined from the tests at static three-point bending of GFRP-foam specimens.

Material	Mean (μ)	Standard Deviation (s)	Coefficient of Variation (δ)%
GFRP-Foam—Bending Strength (MPa)	26.5	3.3	12.452
GFRP-Foam Bending Modulus (GPa)	2.8	0.4	14.285
Core shear ultimate strength (MPa)	0.843	0.105	12.455
Core shear yield stress (MPa)	0.069	0.009	13.043
Facing stress (MPa)	92.8	11.6	12.5

**Table 3:** Accelerated fatigue life testing results of the GFRP-foam specimens.

Specimen No.	The Number of Cycles to Failure in Accelerated Conditions	Accelerated Frequency Level (Hz)
1	20,756	2
2	22,321	2
3	24,759	2
4	26,098	2
5	27,479	2
6	14,389	3
7	16,342	3
8	17,743	3
9	19,902	3
10	21,872	3
11	2763	4
12	3473	4
13	4093	4
14	5764	4
15	6034	4
16	1234	5
17	1456	5
18	1789	5
19	2021	5
20	2341	5

**Table 4:** Determination of the number of cycles to failure for normal testing conditions (1.5 Hz loading frequency) of GFRP-foam specimens.

Specimen No.	The Number of Cycles to Failure in Accelerated Testing Conditions	Accelerated Frequency Level (Hz)	Acceleration Factor	The Number of Cycles to Failure in Normal Testing Conditions
1	20,756	2	2.504	51,973
2	22,321			55,892
3	24,759			61,997
4	26,098			65,349
5	27,479			68,807
6	14,389	3	9.133	131,415
7	16,342			149,251
8	17,743			162,047
9	19,902			181,765
10	21,872			199,757
11	2763	4	22.87	63,190
12	3473			79,428
13	4093			93,607
14	5764			131,823
15	6034			137,998
16	1234	5	46.614	57,522
17	1456			67,870
18	1789			83,392
19	2021			94,207
20	2341			109,123

**Table 5:** The dependence of the reliability indicators as function of the number of cycles to failure in normal testing conditions.

The Number of Cycles to Failure in Normal Testing Conditions	Reliability R (t)	Unreliability F (t)	Pdf f(t) × 10^−6^	Failure Rate λ(t) × 10^−6^
51,973	0.965	0.035	5.67	21.7
55,892	0.917	0.083	6.15	23.8
57,522	0.868	0.132	6.35	24.7
61,997	0.819	0.181	6.85	27.2
63,190	0.770	0.230	6.98	27.9
65,349	0.721	0.279	7.20	29.1
67,870	0.672	0.328	7.44	30.6
68,807	0.622	0.378	7.52	31.2
79,428	0.573	0.427	8.29	37.8
83,392	0.524	0.476	8.48	40.4
93,607	0.475	0.525	8.70	47.4
94,207	0.426	0.574	8.70	47.9
109,123	0.377	0.623	8.32	59.0
131,415	0.327	0.673	6.61	77.5
131,823	0.278	0.722	6.57	77.9
137,998	0.229	0.771	5.96	83.5
149,251	0.180	0.82	4.80	94.0
162,047	0.131	0.869	3.53	106.7
181,765	0.082	0.918	1.95	127.8
